# Are We Compensating for the Lack of Physical Activity in Our Diabetic Patients with Treatment Intensification?

**DOI:** 10.3390/sports5030058

**Published:** 2017-08-07

**Authors:** Maja Cigrovski Berkovic, Ines Bilic-Curcic, Marina Gradiser, Davorka Herman-Mahecic, Vjekoslav Cigrovski, Marul Ivandic

**Affiliations:** 1 Clinical Department of Endocrinology, Diabetes and Metabolism, University Hospital Centre “Sestre Milosrdnice”, 10000 Zagreb, Croatia; maja.cigrovskiberkovic@gmail.com (M.C.B.); dherman_cro@yahoo.com (D.H.-M.); 2 Faculty of Medicine, J. J. Strossmayer University Osijek, Clinical Hospital Center Osijek, 31000 Osijek, Croatia; mivandic@yahoo.com; 3 Department of Internal medicine, General Hospital Čakovec, 40101 Čakovec, Croatia; marina.gradiser@gmail.com; 4 Faculty of Kinesiology, University of Zagreb, 10000 Zagreb, Croatia; vjekoslav.cigrovski@kif.hr

**Keywords:** leisure time physical activity, diabetes mellitus, glycemic control, body mass index, hypoglycemia

## Abstract

Background: We studied the association between leisure time physical activity (LTPA) and glycemic control, body mass index (BMI), and hypoglycemic incidents in type 1 (T1DM) and type 2 diabetes patients (T2DM). Methods: This is a cross-sectional study of 198 diabetic patients (60 with type 1 diabetes, 138 with type 2 diabetes). LTPA was assessed by a validated 12-month questionnaire. Patients were grouped as sedentary and moderately to vigorously active. Outcome measures were Hemoglobin A1c (HbA1c), BMI, and hypoglycemic episodes. Results: LTPA effect on the HbA1c reduction was present in diabetes type 1 patients. Patients who were involved in the moderate to vigorous-intensity physical activity had a greater decrease in the HbA1c (*p* = 0.048) than patients with low physical activity (*p* = 0.085). Level of LTPA was neither associated with increased number of hypoglycemic episodes, nor BMI. After an average of 4 years of diabetes, the number of patients requiring more than one antidiabetic agent increased, although the observed difference did not correlate with LTPA level. Conclusions: LTPA has an influence on the regulation of diabetes type 1, and intensification of medical treatment is compensating for the lack of lifestyle change—especially in type 2 diabetics.

## 1. Introduction

Diabetes is a metabolic disease primarily characterized by abnormalities in glucose metabolism. The pathophysiology is heterogeneous, involving genetic, environmental, and lifestyle factors [1,2]. There have been many developments in the management of diabetes (especially type 2 diabetes) in the last few decades; however, poor glycemic control remains a worldwide problem. Disease is associated with high morbidity and mortality, the development of severe and debilitating vascular complications which translate into socio-economic implications accounts for 10.8% of the total global health care expenditure for diabetes-related health care costs [3,4,5]. When discussing the problem of unmet need for better management of diabetes, we often mention clinical inertia in treatment intensification, which relates to health care practice as well as patients’ barriers (such as fear of treatment side effects, increased regimen complexity, and lack of patient adherence) [6]. However, on the other side, by the addition of new drugs are we forgetting the basics of diabetes treatment such as patient reeducation and life-style changes? It is well known that in the early stages of disease, life-style interventions are as effective or even more efficacious in the prevention of progression from prediabetes to clinically manifest diabetes [7,8]. Later on, structured physical activity has the role of maintaining glycemic control and the prevention of chronic complications [9]. Although nominally important, is the lack of structured physical activity in diabetes treatment the missing link to maintain long-term glycemic control? Data on the role of changes of leisure time physical activity (LTPA) in the treatment of diabetes type 1 and type 2 and its effect on development/progression of chronic complications are limited and conflicting. In this research, we investigated the impact of physical activity on the glycemic control of patients with type 1 and type 2 diabetes, as well as its correlation with other oral treatment options/insulin.

## 2. Patients and Methods

We surveyed a total of 200 patients (60 with type 1 diabetes, 138 with type 2 diabetes, and 2 with gestational diabetes) from different parts of Croatia on a leisure time physical activity (LTPA) questionnaire. The questionnaire was taken from Finn Diane group, translated to Croatian, and then back to English for analysis [10]. Prior to fulfilling the questionnaire, patients were informed about the study and their written consent was obtained. The study was conducted in accordance with the Declaration of Helsinki (2004) and the International Conference on Harmonization Guidelines for Good Clinical Practice (1996). Data on patients’ demographics, duration of diabetes, diabetes drugs/insulin at the point of diabetes diagnosis and at the time of questionnaire fulfillment, and level of glycemic control (HbA1c), education, and employment was known upon data analysis. Final data analysis was performed for 181 patients; separately for 55 patients with type 1 and 126 with type 2 diabetes who had completed the questionnaire fully.

For each of 20 different physical activity types, participants rated how many times each month they engaged in activity, the average duration of activity, and subjective intensity. Each activity was given its metabolic equivalent (MET) value [11]. The total amount of leisure time physical activity was computed as mean frequency × mean duration (h) × subjective intensity × MET value, and it was expressed as MET × h/week. Subjective intensity level ranged from 0 to 3; if it was zero, the amount of physical activity was multiplied by one; if it was two, the amount was multiplied by three; and if it was three, the level was multiplied by four. Patients were classified as sedentary (less than 10 MET h/week), moderately active (10 to 40 MET × h/week), or active (more than 40 MET × h/week) with respect to their level of physical activity [12,13]. Outcome measures were HbA1c, BMI, and hypoglycemic episodes.

Statistical analyses were performed using SPSS Statistics version 22 (IBM Corp. Released 2013. IBM SPSS Statistics for Windows, Version 22.0. Armonk, NY, USA). Continuous variables are given as median with interquartile range due to non-normality of distribution. Categorical variables are given as percentages, and they were analyzed using AUC (area under curve) for estimating effect sizes, and Cramer’s V coefficient as measure of association. Between-group differences in HbA1c reduction were analyzed using Analysis of variance (ANOVA) for normally distributed variables that had homogenous variances. Univariate binary logistic regression was used to estimate effects of LTPA on clinical characteristics of type 1 diabetes mellitus (T1DM) patients. A *p* value of <0.05 was considered statistically significant.

## 3. Results

Overall 181 participants (55 with type 1 and 126 with type 2 diabetes) fully completed the LTPA questionnaire. Median age of diabetes type 1 and type 2 patients was 37, ranging from 15 to 69 years, and 62, ranging from 18 to 80 years, respectively. Average duration of diabetes was 11 years for patients with type 1, and 4 years for type 2 diabetes patients. At the time of diagnosis, average HbA1c was 9.7% and 8.0% for T1DM and T2DM patients, respectively (data are presented in Table 1). A significant proportion of patients with type 2 diabetes was obese (55.1%), while hypoglycemic events were more frequent in T1DM patients (Table 1).

**Figure 1:**
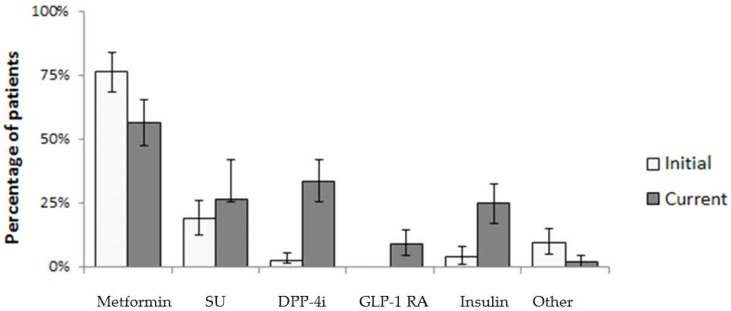
Treatment intensification during course of disease for patients with type 2 DM (*n* = 126); error bars represent 95% confidence interval. SU, sulphonylurea; DPP-4i, dipeptidyl peptidase-4 inhibitors; GLP-1 RA, glucagon-like peptide receptor agonist.

Data on diabetes therapy for patients with T2DM initiated at the time of diagnosis and at the time of the survey are summarized in Table 2. At the time of diagnosis, 76.2% of T2DM patients were treated with metformin (15.9% of patients in combination with other oral antidiabetic agents (OAD), while 19% were treated with sulphonylurea. In 4% of patients, insulin was initial therapy. At the time of survey, with respect to diabetes duration after an average of 4 years, the number of patients requiring more than one antidiabetic agent increased, although the observed difference did not correlate with the level of LTPA (data not shown). On the other hand, at the time of survey, the number of patients treated with insulin (4% vs. 24.6%) increased, as did the number of patients treated with combination therapy excluding insulin (15.5% vs. 44%). In Figure 1, treatment intensification according to specific class of medication is presented.

Current involvements in LTPA of participants are shown in Table 3. Type 2 diabetes patients were usually involved in low-intensity physical activity, with a decrease of physical activity in the last 10 years. On the other hand, patients with type 1 diabetes further increased level of LTPA in the same period of time and were involved in moderate- to high-intensity activity to a greater extent.

LTPA effect on the HbA1c reduction was present only in type 1 diabetes patients (Table 4), while in type 2 patients no effect whatsoever was noted (data not shown). Patients who were involved in moderate to vigorous intensity physical activity had a greater decrease in the HbA1c level (from 11.2% to 7.3%, *p* = 0.048) than patients whose physical activity was of low intensity (from 8.1% to 7.0%, *p* = 0.085). In addition, patients with the greatest increase in LTPA had the most prominent reduction in HbA1c (F_(2,26)_ = 4.319, *p* = 0.025).

However, there was no association between LTPA and HbA1c levels or BMI in either group of patients. Additionally, no correlation of LTPA with various treatment options defined as monotherapy, combination therapy including oral antidiabetics, or insulin therapy was established (data not shown) in diabetes type 2 patients. In both type 1 and type 2 diabetes, the level of LTPA was not associated with an increased number of hypoglycemic episodes.

## 4. Discussion

In our study, LTPA had no significant influence on glucose regulation in type 2 diabetes patients. This could be a result of therapy intensification in those individuals unable to implement significant lifestyle changes, ultimately leading to polypharmacy in order to achieve therapeutic goals, resulting in increase of health care costs. For example, in the LOOK AHEAD study, intensive lifestyle intervention (ILI) participants required less medication while improving their glucose and lipid regulation, but at the same time patients in the general lifestyle recommendation arm (DSE) had more modest improvements in these parameters with an increase in medication use [14]. Although no difference in cardiovascular risk reduction was seen after almost 8-year follow-up between ILI and DSE groups, an unquestionable benefit was seen in weight reduction, use of less medication, and improvement in quality of life in the ILI treatment arm. The real question is, are we compensating for the lack of lifestyle intervention in diabetic patients with treatment intensification? 

Data suggest that engaging in LTPA may be associated with improved survival in participants with diabetes and poor self-rated physical health status [15].

Previous studies have shown that moderate-to-vigorous LTPA was associated with the resolution of metabolic syndrome and the improvement of components of metabolic syndrome including insulin resistance and hyperglycemia, but no changes were seen with low-intensity LTPA [10]. In addition, large meta-analyses have confirmed a beneficial role of moderate- to high-intensity LTPA in diabetes prevention [16]. However, those studies included patients with metabolic syndrome, but without diabetes type 2 who were not treated with antihyperglycemic agents as were in our survey; therefore, it was possible to more accurately establish a relationship between LTPA and hyperglycemia.

Furthermore, in the Italian Diabetes and Exercise Study, only the supervised exercise intervention strategy was effective in improving HbA1c and cardiovascular risk profile, but counseling alone had no effect on glucose regulation [17]. In a Japanese cohort study, the amount of LTPA required to lower HbA1c appeared to be greater than that required to improve other cardiovascular risk factors [18]. This agrees with our results, as it seems that without supervised structured exercise program we cannot achieve significant improvements in the glucose regulation as daily physical activity is not sufficient to overcome insulin resistance and does not have sufficient impact on hyperglycemia. However, in clinical practice it is difficult to measure and quantify the physical activity—specifically structured physical activity—if not performed under supervision. This was a retrospective study; questionnaires were filled based on patients’ memory, and the number of patients included was quite small. Thus, it is difficult to estimate whether LTPA had some kind of impact on the development of diabetes type 2, and the extent of its impact on glucoregulation. There also remains the question of nutrition, since our questionnaire was based solely on LTPA. If there is no adequate diet intervention, then the effect of LTPA on diabetes regulation is diminished or incapacitated. On the other hand, in surveyed type 1 diabetes patients we have seen the greatest HbA1c reduction in those involved in the moderate-to-vigorous intensity physical activity, with no difference in the occurrence of hypoglycemia or significant impact on BMI. Current literature on the effects of LTPA on the glucoregulation of patients with type 1 diabetes is controversial [19]. Some studies showed beneficial effects of PA on glucoregulation and HbA1c decrease [20,21], while others demonstrated no improvement in glycemic control after physical activity [22]. Another issue is the effect of LTPA on chronic complications of hyperglycemia in both type 1 and type 2 diabetes [23]. Those results and their interpretation are even more complex. For example, in Japanese subjects with type 2 diabetes, unlike in Western diabetic populations, LTPA of 15.4 MET h/week was associated with a significantly lower risk of stroke and total mortality, but independently of cardiovascular risk factors or events [24]. In addition, there is growing body of evidence linking obesity and impairment of cognitive function, even in middle aged people [25,26,27]. Thus, it is reasonable to assume that limitations in cognitive function could influence the lower level of LTPA in the T2DM group of patients, mainly consisting of overweight and obese individuals.

## 5. Conclusions

In conclusion, in order to achieve a significant impact on glucose regulation and cardiovascular risk factors, the implementation of a structured exercise program in combination with appropriate diet is necessary. Moreover, by intensification of medical treatment we are trying to compensate for the lack of lifestyle change, thus contributing to polypharmacy. Long-term consequences are increased medical costs, more side effects, lack of compliance, and increase in adverse drug interactions. Therefore, the cost of implementing intensive lifestyle intervention programs adjusted for the specific needs of type 1 and type 2 patients would be fully justified in order to reduce the costs of diabetes and affiliated cardiovascular risk factors treatment and ultimately to prevent the development of chronic complications.

## Figures and Tables

**Table 1:** Baseline characteristics of study participants (*n* = 181).

Variable	DM Type 1 (*n* = 55)		DM Type 2 (*n* = 126)		*p*	Effect Size
Age (years)	37	(26–44)	62	(54–69)	<0.001	0.93 *
Age at diagnosis (years)	23	(13–29)	51	(45–59)	<0.001	0.97 *
Duration of diabetes (years)	11	(5–19)	4	(8–14)	0.040	0.61 *
HbA1c (%)						
at diagnosis	9.7	(6.9–12.1)	8.0	(7.2–9.9)	0.104	0.60 *
at the time of enrolment	7.0	(6.3–7.6)	6.6	(6.1–7.2)	0.020	0.65 *
Body mass index (kg/m^2^)						
normal (≤24.99)	32	(84.2%)	16	(15.0%)	<0.001	0.65 ^†^
overweight (25.00–29.99)	2	(5.3%)	32	(29.9%)		
obese (≥30.00)	4	(10.5%)	59	(55.1%)		
Hypoglycemia	49	(92.5%)	45	(37.2%)	<0.001	0.51 ^†^
Blood glucose level at hypoglycemia (mmol/L)	3.2	(2.8–3.8)	3.2	(3.0–3.8)	0.757	0.52 *

Data are presented as median (interquartile range) if not stated otherwise; * Area under the curve (AUC); ^†^ Cramer’s V (φ_c_). DM: diabetes mellitus.

**Table 2:** Medication prescribed to participants with type 2 diabetes at the time of diagnosis and at the time of survey.

At the Time of Diagnosis	*n* = 126	(%)	At the Time of Survey	*n* = 126	(%)
Metformin	96	(76.2)	Metformin	71	(56.3)
Sulphonylurea	24	(19.0)	SU	33	(26.2)
DPP-4i	3	(2.4)	DPP-4i	42	(33.3)
GLP-1	0	(0.0)	GLP-1 RA	11	(8.7)
Insulin	5	(4.0)	Insulin	31	(24.6)
Other	12	(9.5)	Other	2	(1.6)
**Complete Therapy Structure**			**Complete Therapy Structure**		
MET	77	(61.1)	MET	22	(17.4)
MET + SU	13	(10.3)	MET + SU	7	(5.6)
SU	5	(3.9)	SU	2	(1.5)
MET + TZD	1	(0.7)	MET + DPP-4i	20	(15.9)
SU + MET + DPP-4i	3	(2.3)	SU + MET + DPP-4i	14	(11.1)
Insulin	3	(2.3)	Insulin	29	(23.0)
AGI	1	(0.7)	SU + DPP-4i	4	(3.1)
MET + SU + insulin	2	(1.5)	DPP-4i	3	(2.3)
Repaglinide	2	(1.5)	OAD + GLP-1 RA	9	(6.8)
Not specified OAD	3	(2.3)	Not specified OAD	2	(1.5)
SU + MET + TZD	1	(0.7)	GLP-1 RA + insulin	2	(1.5)

MET, metformin; SU, sulphonylurea; TZD, thiazolidinedione; AGI, alpha-glucosidase inhibitors; DPP-4i, dipeptidyl peptidase-4 inhibitors; GLP-1 RA, glucagon-like peptide receptor agonist.

**Table 3:** Indicators of current leisure physical activity (PA) by diabetes type.

Type of PA	Type 1 *n* = 55 (%)		Type 2 *n* = 126 (%)		*p*	Effect Size
Change of PA during the last 10 years						
Decrease	20	(37.0)	65	(51.6)	<0.001	0.30 ^†^
No change	7	(13.0)	35	(27.8)		
Increase	27	(50.0)	26	(20.6)		
Low intensity PA	35	(63.6)	101	(80.2)	0.018	0.18 ^†^
At least 3 times a week ≥ 60 min of moderate-to-vigorous PA	20	(36.4)	25	(19.8)		
Total	55	(100.0)	126	(100.0)		

Data are presented as number (percentage) of respondents if not stated otherwise; IQR, interquartile range; ^†^ Cramer’s V (φ_c_).

**Table 4:** Effects of leisure time PA (LTPA) on clinical parameters of T1DM patients (*n* = 55).

Leisure Time Physical Activity							
Variable	At Least 3 Times a Week ≥60 min of Moderate-to-Vigorous PA		Less PA		OR	(95% CI)	*p*
Average age (years)	31	(26–43)	38	(26–45)	0.97	(0.92–1.03)	0.282
Age at diagnosis (years)	21	(13–29)	23	(12–29)	1.01	(0.96–1.06)	0.771
Average duration of diabetes	11	(6–15)	10	(5–22)	0.99	(0.93–1.06)	0.809
HbA1c (%)							
at diagnosis	11.2	(8.5–13.4)	8.1	(6.5–11.8)	1.27	(0.97–1.65)	0.085
at the time of enrolment	7.3	(6.7–8.6)	7.0	(6.2–7.5)	1.87	(1.00–3.45)	0.048
Average body mass index (kg/m^2^)	19	(16–21)	21	(18–24)	0.95	(0.85–1.06)	0.359
Number of patients experiencing hypoglycemia							
no	1	(25.0)	3	(75.0)	1		
yes	19	(38.8)	30	(61.2)	1.90	(0.18–19.6)	0.590
Blood glucose level at hypoglycemia (mmol/L)	3.6	(2.8–3.9)	2.9	(2.9–3.6)	1.72	(0.56–5.26)	0.344

Data are presented as median (interquartile range) if not stated otherwise; PA, physical activity; OR, odds ratio, univariate binary logistic regression; 95% CI, 95% confidence interval; *p*, statistical significance of the OR.
